# Ultra-High-Resolution Liquid Chromatography Coupled with Electrospray Ionization Quadrupole Time-of-Flight Mass Spectrometry Analysis of *Tessaria absinthioides* (Hook. & Arn.) DC. (Asteraceae) and Antioxidant and Hypocholesterolemic Properties

**DOI:** 10.3390/antiox13010050

**Published:** 2023-12-28

**Authors:** Mariana Rey, María Sol Kruse, Jessica Gómez, Mario J. Simirgiotis, Alejandro Tapia, Héctor Coirini

**Affiliations:** 1 Laboratorio de Neurobiología, Instituto de Biología y Medicina Experimental (IBYME-CONICET), Ciudad Autónoma de Buenos Aires C1428ADN, Argentina; sol.kruse@conicet.gov.ar; 2 Instituto de Biotecnología-Instituto de Ciencias Básicas, Universidad Nacional de San Juan (UNSJ), San Juan J5400ARL, Argentina; jegomez@unsj.edu.ar (J.G.); atapia@unsj.edu.ar (A.T.); 3 Consejo Nacional de Investigaciones Científicas y Técnicas (CONICET), Ciudad Autónoma de Buenos Aires C1425FQB, Argentina; 4 Instituto de Farmacia, Facultad de Ciencias, Campus Isla Teja, Universidad Austral de Chile, Valdivia 5090000, Chile; mario.simirgiotis@uach.cl; 5 Center for Interdisciplinary Studies on the Nervous System (CISNe), Universidad Austral de Chile, Valdivia 5090000, Chile

**Keywords:** UHPLC-ESI-QTOF-MS phenolic compound analysis, antioxidant, di-caffeoylquinic acid, natural compounds, cholesterol

## Abstract

Recently, we reported the chemical profile and the hypocholesterolemic effects of a decoction of *Tessaria absinthioides* (Hook. & Arn.) DC. (Asteraceae). In this study, we evaluated a methanolic extract (METa) instead. Metabolite profiling was conducted using ultra-high-resolution liquid chromatography coupled with electrospray ionization quadrupole time-of-flight mass spectrometry (UHPLC-ESI-QTOF-MS), identifying thirty compounds, including flavonoids, phenolic acids, fatty acids, and phorbolesters. Antioxidant properties were assessed through 2,2-diphenyl-1-picrylhydrazyl (DPPH), Trolox equivalent antioxidant activity (TEAC), ferric-reducing antioxidant power (FRAP), and inhibition of lipid peroxidation in erythrocytes (ILP) assays, exhibiting robust antioxidant activity. The in vivo impact of METa on serum lipid parameters and liver X receptors (LXRs) was evaluated in a hypercholesterolemic animal model. After 14 days on a high-fat diet, male rats received either a vehicle (V) or METa_100_, METa_200_ or METa_500_ (100; 200 and 500 mg METa/kg animal, respectively) for an additional two weeks. METa_500_ reduced total cholesterol levels (17.62%; *p* < 0.05) and all doses increased high-density lipoprotein cholesterol levels (METa_100_: 86.27%; METa_200_: 48.37%, and METa_500_: 29.42%; *p* < 0.0001). However, METa did not alter LXRs expression. The observed antioxidant and hypocholesterolemic properties of METa may be linked to the presence of six di-caffeoylquinic acids. These findings underscore *T. absinthioides* as a potential candidate for the treatment of metabolic disease.

## 1. Introduction

*Tessaria absinthioides* (Hook. & Arn.) DC. (Asteraceae), a medicinal species, is one of the South American plants whose chemistry has been consistently updated by numerous authors. Compounds exhibiting various biological activities and their isolation have been documented in prior studies [1,2,3,4]. These studies underscore the presence of flavonoids, fatty acids, sesquiterpenes, and phenolic acids in extracts of both polar and non-polar natures from *T. absinthioides*. We previously reported the chemical profile of a decoction of *T. absinthioides* through ultra-high-resolution liquid chromatography Orbitrap MS analysis (UHPLC–PDA–OT-MS/MS), revealing the presence of fifty-one compounds, twelve of which were reported for the first time [5]. Among these compounds, several phenolics and flavonoids have been reported as free radical scavengers, antioxidants, lipid peroxidation inhibitors, antitumor agents, hepatoprotectors, and more, with the potential to improve human health [6,7,8]. The quest for polyphenol antioxidants or free radical scavengers in medicinal plants and other sources has been growing, primarily driven by the essential and beneficial role these chemical compounds play in the human diet. They contribute to the prevention of diseases or pathologies associated with oxidative stress and help maintain overall human health [9]. Several studies have reported that members of the Asteraceae family are capable of exhibiting antioxidant properties through different pathways [6,7,10]. Also, a recent study has reported that a lyophilized decoction of *T. absinthioides* from Argentina and Chile exhibits free radical scavenging and antimicrobial activity [4].

The consumption of high-fat diets has been linked to the development of health problems [11]. Some drugs used in the treatment of hypercholesterolemia may lead to secondary effects or prove ineffective in certain patients [12,13]. Therefore, the scientific exploration of traditional medicinal plants represents an opportunity to investigate new therapeutic options. Some members of the Asteraceae family have shown hypocholesterolemic properties, even when administered for short term periods [14]. Numerous reports have highlighted that plant compounds, including phytosterols, diterpenes, phenolic acids and flavonoids, can effectively reduce serum lipids and cholesterol levels while modulating the expression of liver X receptors (LXRs) [15,16]. These receptors play a pivotal role in cholesterol metabolism [17] and are involved in glucose and fatty acid metabolism, as well as processes related to the immune system, inflammation and steroidogenesis [18]. The LXRα subtype is expressed in the liver, kidney, small intestine, adipose tissue and macrophages, while the LXRβ subtype is ubiquitously expressed [19]. Both LXR subtypes can induce hepatic lipogenesis, with LXRα being the predominant subtype in the hepatic pathway [20].

In a previous work, we demonstrated that a 6-week administration of a decoction of *T. absinthioides* reduces total cholesterol (TC) levels, increases high-density lipoprotein-c (HDL-c) levels and modifies the expression of LXRs in animals fed a high-fat diet [5].

Building on these promising results, we set a study to investigate whether a concentrate of this plant could enhance the observed effects. The objectives of this study were to analyze the full metabolome polyphenolic profile, assess the total phenolics and flavonoids content and determine antioxidant capacity in vitro as well as the hypocholesterolemic properties in vivo.

Our hypothesis posits that the compounds in METa produce antioxidant effects and may enhance lipid profile levels while modulating LXRs expression in animals on a high-fat diet. These findings will provide additional insights into the beneficial properties of *T. absinthioides*, supporting its potential as a source of new antioxidant and hypocholesterolemic compounds.

## 2. Materials and Methods

### 2.1. Chemicals

Ultra-pure water (<5 µg/L total organic carbon, TOC) was obtained from a water purification system Ari-um 126 61316-RO plus and Arium 611 UV unit (Sartorius, Goettingen, Germany). Methanol (HPLC grade) and formic acid (puriss. p.a. for mass spectrometry) were obtained from J. T. Baker (Phillipsburg, NJ, USA). Chloroform (HPLC grade) was obtained from Merck (Santiago, Chile). The only HPLC standards available used were rhamnacin, sakuranetin, genkwanin, isorhamnetin, 2-Hydroxyoctadecanoic acid and hesperetin, with purity higher than 95% by HPLC (Sigma-Aldrich Chem. Co., St Louis, MO, USA or Extrasynthèse, Genay, France).

### 2.2. Botanical Description, Plant Material and Methanolic Extract of T. absinthioides (METa)

*T. absinthioides*, commonly known as “pájaro bobo”, “suncho”, or “brea” (Asteraceae), is a shrub that reaches a height of 1–1.5 m. It is found in Bolivia, Chile, Uruguay and Argentina, including the Cuyo Region. The plant thrives in humid and sandy soils along the banks of rivers, mountain streams and irrigation ditches. The species is characterized by a stem with a beige/brown pale shoot color and a tomentose indumentum. The leaves have a tomentose indumentum, and the margin is serrated. The inflorescence has a pubescent receptacle. The flowers have lobes with a length ranging from short to 1 mm, and the margin of the corolla lobes is not thickened. The number of internal flowers ranges from 5 to 15. The fruit has a glabrous indumentum on the cypsela. The naturally grown *T. absinthioides* was collected in the locality “Médano de Oro”, Rawson district, San Juan, Argentina, during the conditioning process of a farm. The aerial parts of *T. absinthioides* were dried at room temperature (25 °C) and stored in the absence of light and heat. A voucher specimen was deposited in the “Laboratorio Productos Naturales of the Universidad Nacional de San Juan” (voucher number IBT-ATTa-2022).

The METa was prepared from the air-dried ground aerial parts of the plant (1 Kg) by extracting under re-flux with methanol (3000 mL) three times for 30 min each at 60 °C. The mixtures were concentrated under reduced pressure in a Yamato rotary evaporator (RE-300-AW2, Santa Clara, CA, USA). The METa (100 g, 10% *w*/*w* yield in terms of dry starting material) was stored at −20 °C until use.

### 2.3. Ultra-High-Resolution Liquid Chromatography Analysis (UHPLC-ESI-QTOF-MS)

The separation and identification of secondary metabolites from METa were conducted using a UHPLC-ESI-QTOF-MS system, which was equipped with UHPLC Ultimate 3000 RS featuring Chromeleon 6.8 software (Dionex GmbH, Idstein, Germany), along with a Bruker maXis ESI-QTOF-MS and the software Data Analysis 4.0 (all Bruker Daltonik GmbH, Bremen, Germany). The chromatographic equipment included a quaternary pump, an autosampler, a thermostated column compartment, and a photodiode array detector.

The elution was performed using a binary gradient system with eluents A (0.1% formic acid in the water) and B (0.1% formic acid in acetonitrile). The gradient started as follows: isocratic 1% B (0–2 min), 1–5% B (2–3 min), isocratic 5% B (3–5 min), 5–10% B (5–8 min), 10–30% B (8–30 min), 30–95% B (319–38 min) and 1% B isocratic (39–50 min). The separation was carried out with an acclaim Thermo 5 µm C18 80 Å (150 mm × 4.6 mm) column at a flow rate of 1.0 mL/min. ESI-QTOF-MS experiments in negative ion mode were recorded and the scanning range between 100 and 1200 *m*/*z*. Electrospray ionization (ESI) conditions included capillary temperature of 200 °C, a capillary voltage of 2.0 kV, a dry gas flow of 8 L/min and a nebulizer pressure of 2 bars. The experiments were performed in automatic MS/MS mode. The structural characterization of the bioactive compounds was based on high resolution mass spectrometry (HR-MS), fragmentation patterns, and with data from the literature [4]. For the analysis, 5 mg of METa was dissolved in 2 mL of methanol and passed through a polytetrafluoroethylene filter. Ten µL of the mixture was injected into the apparatus. MS data were analyzed using the software’s Bruker Data Analysis 4.0 (Bruker Daltonik GmbH, Bremen, Germany) and ACD lab spectrum processor (New York, NY, USA).

### 2.4. The Total Phenols Assay by Folin−Ciocalteu Reagent (FCR) and Flavonoids Content

The total phenolics content, expressed as milligrams of gallic acid equivalents (GAE) per gram of METa (mg GAE/g METa), was determined with a total phenols assay with FCR, following the protocol described by [4,21]. On the other hand, the flavonoids content in METa, expressed as milligrams of quercetin equivalents (QE) per gram of METa (mg QE/gMETa), was determined with an AlCl_3_ assay following the protocol described by [4]. The values were obtained using a Multiskan FC Microplate Photometer (Thermo Scientific, Waltham, MA, USA). Both tests were carried out in quadruplicate and results are expressed as mean ± SD.

### 2.5. In Vitro Antioxidant Activity

#### 2.5.1. Radical Scavenging Capacity Assay of 2,2-Diphenyl-1-picrylhydrazyl (DPPH)

The free radical scavenging effect of METa was evaluated by DPPH assay. A freshly prepared DPPH methanolic solution at 20 mg/L was used for the assays. Reaction mixtures containing METa and the reference compound catechin at concentrations ranging from 1 to 100 μg/mL were dissolved in methanol and mixed with the DPPH methanolic solution at 20 mg/L (0.05 mM or 50 µM). The mixtures were then incubated at 37 °C for 30 min in microtiter plates. The scavenging activities were evaluated spectrophotometrically at 517 nm, with the absorbance (Abs) of the DPPH radical serving as a reference. A decrease in color intensity indicated the free radical scavenging efficiency of METa or catechin. DPPH antioxidant capacity was calculated as follows:

% scavenging effect = [1 − (Abs-sample − Abs-blank)/Abs-DPPH] × 100

The concentration providing 50% of radical scavenging activity (EC_50_) was determined from the graph depicting inhibition percentage at 517 nm against the METa concentration. Catechin (≥98%, Sigma-Aldrich, St. Louis, MO, USA) was used as a reference compound (EC_50_ 4.1 μg/mL). The tests were carried out in quadruplicate.

#### 2.5.2. Ferric-Reducing Antioxidant Power Assay (FRAP)

The FRAP assay was conducted in a microplate, following a method that has been previously reported [4]. In brief, the FRAP reagent was mixed with a methanolic solution of METa (1 mg/mL). Simultaneously, a calibration curve was prepared by combining the FRAP reagent with Trolox solutions from 0 to 1 mmol/L concentrations. The Abs values of the mixtures were measured at 593 nm using a Multiskan FC Microplate Photometer (Thermo Scientific, Waltham, MA, USA). Results were obtained through linear regression from the FRAP–Trolox calibration plot and are expressed in equivalent milligrams of Trolox per gram of METa (mg Trolox/g METa). The tests were carried out in quadruplicate.

#### 2.5.3. Trolox Equivalent Antioxidant Activity Assay (TEAC)

The TEAC assay was conducted following the method described in [4] with minor modifications. In brief, 10 µL of METa (1 mg/mL) or Trolox standard was combined with 200 µL of ABTS^•+^ (dissolved in phosphate buffered saline, PBS). The mixture was stirred with a vortex for 10 s, and the Abs at 734 nm was measured after a 4 min reaction at 30 °C. The results were determined by linear regression from a calibration plot constructed with Trolox concentrations ranging from 0 to 1 mM and are expressed in equivalent milligrams of Trolox per gram of METa (mg Trolox/g METa). The tests were carried out in quadruplicate.

#### 2.5.4. Inhibition of Lipid Peroxidation (ILP) in Erythrocytes

The ILP assay in erythrocytes was conducted following a method that has been previously reported [4]. The inhibitory potential of two METa concentrations (100 and 250 µg METa/mL) and the reference compound catechin (100 µg/mL) against erythrocyte lipoperoxidation induced by tert-Butyl hydroperoxide was determined. The values obtained are expressed as percentages of ILP. The tests were carried out in quadruplicate.

### 2.6. Animals, Diets and Experimental Procedure

Adult male Sprague–Dawley rats (2-month-old; 300–400 g; n = 65–70) were housed under standard laboratory conditions in a temperature and humidity controlled vivarium with a 12 h light/dark cycle and ad libitum access to food and water. All procedures concerning animal care and use were carried out according to the European Community Council Directive (86/609/EEC) and the guidelines of the National Institutes of Health Guide for the Care and Use of Laboratory Animals and approved by the ethical committee of IBYME (25/03/2021) in CABA, Argentina.

The animals were subjected to a normal diet (3.3 kcal/g, protein (18.2%), carbohydrates (56.9%), lipids (3.9%), vitamins and minerals (3%), fiber (5%) and humidity (13%); Gepsa feeds, Grupo Pilar SA, Argentina) from 21 days old to 60 days old. Then, the animals were equally divided into two groups. One group was subjected to an experimental high-fat diet (HFD) to induce a hypercholesterolemic state, whereas the other group remained with the same normal diet (ND) [22]. The high-fat diet was prepared as we described previously (4.8 kcal/g; normal diet (60%) + bovine refined fat (38%; Productos Reciento, Dinamarg SA, Argentina) + cholesterol (2%; sc-202539S, Santa Cruz Biotechnology, Dallas, TX, USA)) [5,22].

After 2 weeks, the animals received the supplementation with three different doses of METa (METa_100_ (100 mg METa/Kg animal), METa_200_ (200 mg METa/Kg animal) or METa_500_ (500 mg METa/Kg animal)) or V (2% ethanol 100°; 2% propylene glycol and 10% tween in hexadistilled water). The METa stock solution concentration was 0.5 g/L. Thus, the animals (n = 40) were divided into eight groups: HFDV, HFDMETa_100_, HFDMETa_200_, HFDMETa_500_, NDV, NDMETa_100_, NDMETa_200_ and NDMETa_500_ (n = 5–7 in each one). The supplementation was administered daily with an intragastric probe (gavage) for 14 days. The body weight of the animals (BW) was recorded weekly between 09:00 and 10:00 a.m. from the beginning of the experiment. The BW gain was determined as the difference between the BW after 7 or 14 days of METa or V supplementation and the BW on week 2 of the high-fat diet.

The daily food intake was measured at 10:00 a.m. throughout the V or METa supplementation, which lasted for 14 days. Following this period, the animals, fasted for 6 h, were rendered unconscious by CO_2_, and then decapitated. Trunk blood was collected to determine the lipid parameters. The liver was dissected, frozen and stored at −80 °C [5,22].

The HFDMETa_100_, HFDMETa_200_ and HFDMETa_500_ were compared to HFDV. The supplementation with METa did not cause any effect in the animals fed with the normal diet.

#### 2.6.1. Lipid Profile Parameters

The levels of TC, high-density lipoprotein cholesterol (HDL-c), low-density lipoprotein-c (LDL-c) and TG were determined in blood samples as we previously described [5,22].

#### 2.6.2. LXRs Protein Expression

The expression of LXRs was quantified by Western blot in homogenates of liver [5,22]. The primary antibodies were anti-LXRα (rabbit, 1:1000) and anti-LXRβ (goat, 1:1000). The primary antibody anti-Actin (goat, 1:3000) was used as protein loading control. The protein expression was referred as percentage of HFDV in each tissue. All the antibodies were obtained from Santa Cruz Biotechnology, Dallas, TX, USA.

### 2.7. Statistical Analysis

The data from different antioxidant assays in vitro were analyzed using InfoStat (version 202l, Universidad Nacional de Córdoba, Córdoba, Argentina).

The data from assays in vivo were statistically analyzed using the commercial software GraphPad Prism (v.4, GraphPad Software Inc., La Jolla, CA, USA), Stat view (v5.0.1, SAS Institute Inc., Hong Kong, China) or SPSS (v.21, IBM SPSS Statistics, Chicago, IL, USA). The significant differences between BW, BW gain, food intake, the levels of the lipid parameters and the expression of LXRs were determined by one-way ANOVA with the factor treatment (V, METa_100_, METa_200_ and METa_500_) followed by the Newman–Keuls’ post hoc test. For all the cases, the data were expressed as mean ± SD and differences were considered significant at *p* < 0.05.

## 3. Results

### 3.1. Metabolite Profiling: UHPLC-ESI-QTOF-MS Analysis of METa

**Figure 1:**
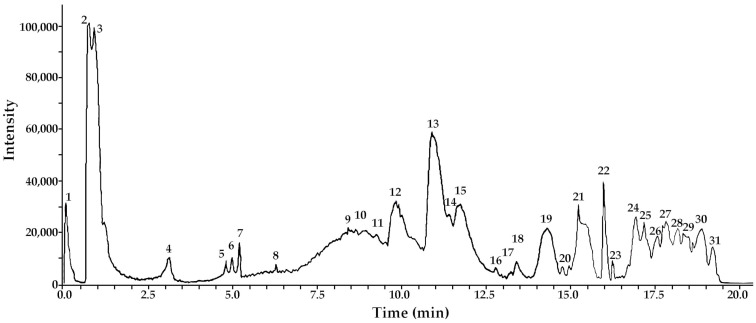
UHPLC Q-TOF-TIC chromatogram of METa. The peak numbers correspond to: internal standard naformiate (**1**); di-caffeoyl quinic acid (cynarin); di-caffeoyl quinic acid 1,5 di C-QA (**2**); di-caffeoyl quinic acid 3,5 di C-QA (**3**); di-caffeoyl quinic acid 4,5 di C-QA (**4**); di-caffeoyl quinic acid 1,3 di C-QA (**5**); di-caffeoyl quinic acid 1,4 di C-QA (**6**); di-caffeoyl quinic acid 1,5 di C-QA (**7**); tessaric acid derivative (**8**); tessaric acid (**9**); 5-Acetyl, 3-hydroxy-4 dihydrocostic acid (**10**); axillarin (**11**); 3-O-Caffeoyl-5-O-malonylquinic acid (**12**); gamma Costic acid (**13**); rhamnacin (**14**); hesperetin (**15**); isorhamnetin (**16**); irigenin (**17**); europetin 7-O-methylmyricetin (**18**); cirsiliol (**19**); arcapillin (**20**); sakuranetin (**21**); genkwanin (**22**); eupatorin (3′,5-Dihydroxy-4′,6,7-trimethoxyflavone; **23**); 2-Hydroxyoctadecanoic acid (**24**); eupatorin 5-methyl eter (**25**); eudesma-2,4(15),11(13)-trien-12-oic acid (**26**); palmitic acid (**27**); cryptocaryol D (**28**); 4 alpha-Phorbol 12,13-didecanoate (**29**); 4 alpha-Phorbol 2,13-didecanoate (**30**) and 4alpha-Phorbol2,12-didecanoate (**31**).

The analysis revealed the presence of thirty compounds in METa, including phenolic acids, phorbolesters, flavones, eudesmane sesquiterpenoids and fatty acids. To the best of our knowledge, some of these compounds are reported for the first time in this species. Identification was achieved through spiking experiments with available standards. Additionally, an access strategy to several databases (MONA mass spectrometry, metaboscape, etc.) and the use of specific software, such as Bruker maXis ESI-QTOF-MS and ACD lab spectrums processor [4,5]. These compounds include twelve flavonoids (peaks 11, 14, 15, 16, 17, 18, 19, 20, 21, 22, 23 and 25), eight phenolic acids (peaks 2, 3, 4, 5, 6, 7, 8 and 12), five terpenes (9, 10, 13, 26 and 28), two fatty acids (peaks 24 and 27) and three phorbolesters (peaks 29, 30 and 31; Figure 1). The complete metabolome identification is shown in Figure 1 and Table 1.

### 3.2. In Vitro Assays: Total Phenolics and Flavonoids Contents and Antioxidant Activity of METa

METa contained high levels of total phenolics, reaching 251.68 mg GAE/g METa. From it, approximately 46% corresponds to flavonoids (116.34 mg QE/g METa). METa displayed robust antioxidant activity, including strong DPPH scavenging with an EC_50_ of 25.72 µg/mL. Additionally, METa exhibited significant ILP in erythrocytes, reaching 60% at a concentration of 100 µg METa/mL. This antioxidant activity was compared with the reference compound catechin, which showed a 74% inhibition at 100 µg/mL (Table 2).

### 3.3. In Vivo Assay

#### 3.3.1. Animals’ BW Gain and Intake of Food

**Figure 2:**
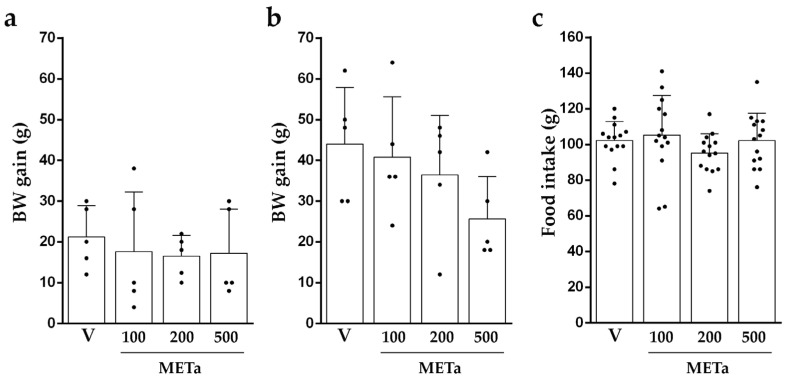
BW gain after 7 days (**a**) or 14 days (**b**) and the average intake of food in 14 days (**c**) of supplementation with V, METa_100_, METa_200_ or METa_500_. Results are METa expressed as mean ± SD from 2–3 independent assays (n = 5 animals/group). Significant differences were determined by one-way ANOVA for BW gain (F_7days_ (3,16) = 0.212; *p* = 0.8863 and F_14days_(3,16) = 1.755; *p* = 0.1963) and food intake (F(3,52) = 1.075; *p* = 0.3680).

The analysis of the BW gain after 7 and/or 14 days of METa supplementation and the food intake did not reveal significant differences between the groups (Figure 2). The BW gain between HFDV and NDV did not differ among groups (*p* = 0.2066). However, HFDV consumed less food than NDV (33.15%; *p* < 0.0001).

In the animals fed with a normal diet, the METa supplementation did not affect either the BW gain after 7 (6–33 g; F(3,16) = 2.742; *p* = 0.0774) or 14 days (16–49 g; F(3,16) = 3.113; *p* = 0.0557) or the food intake (141–153 g; F(3,52) = 0.850; *p* = 0.4729).

#### 3.3.2. Levels of TC, HDL-c, LDL-c and TG

**Figure 3:**
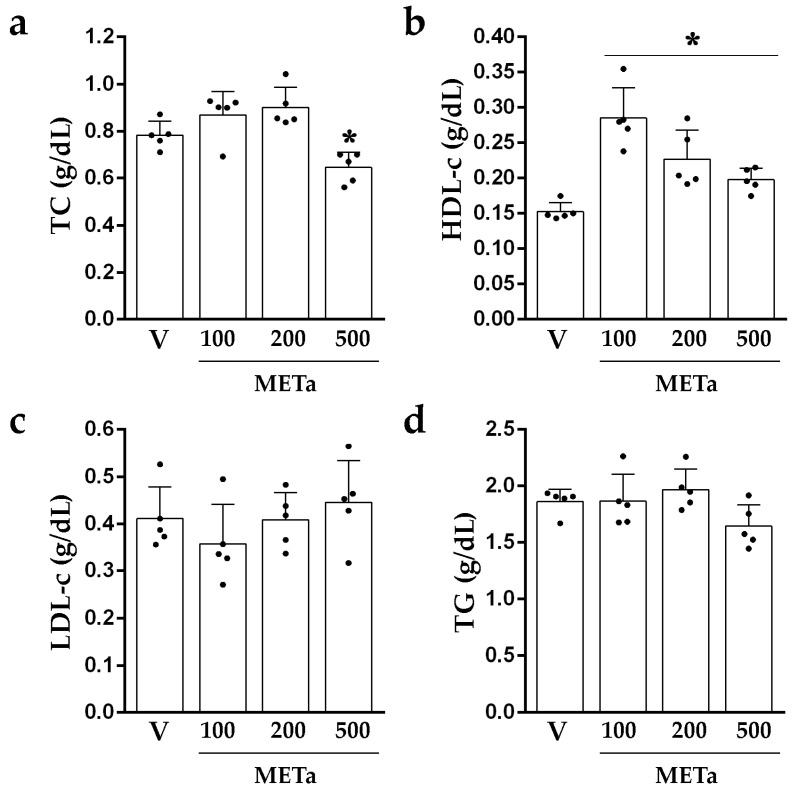
Lipid parameter levels of TC (**a**), HDL-c (**b**), LDL-c (**c**) and TG (**d**) after 14 days of supplementation with V, METa_100_, METa_200_ or METa_500_. Results are METa expressed as mean ± SD from 2–3 independent assays (n = 5 animals/group). Significant differences were determined by one-way ANOVA (F_TC_(3,16) = 10.609; *p* = 0.0004; F_HDL-c_(3,16) = 15.660; *p* < 0.0001; F_LDL-c_(3,16) = 1.161; *p* = 0.3555 and F_TG_(3,16) = 2.699; *p* = 0.0805). * refers to HFDV. * *p* < 0.05, Newman–Keuls’ post-hoc test.

The analysis of lipid parameters revealed significant differences in the levels of TC and HDL-c. HFDE_500_ exhibited lower levels of TC compared to HFDV (17.62%; *p* < 0.05; Figure 3a). In terms of the levels of HDL-c, HFDMETa_100_, HFDMETa_200_ and HFDMETa_500_ presented higher values than HFDV (METa_100_:86.27%; METa_200_: 48.37% and METa_500_: 29.42%; *p* < 0.0001; Figure 3b). The comparison of LDL-c and TG levels did not reveal significant differences between the groups (Figure 3c,d).

The HFDV presented higher levels of TC (44.20%; *p* = 0.0010), LDL-c (75.64% *p* = 0.004) and TG (25.81%; *p* = 0.0068) and lower levels of HDL-c (41.60%; *p* = 0.0029) than NDV. In the animals fed with a normal diet, the METa supplementation did not produce effects on the lipid parameters TC (0.387–0.668 g/dL; F(3,16) = 2.507; *p* = 0.0959), HDL-c (0.157–0.319 g/dL; F(3.16) = 2.103; *p* = 0.1401), LDL-c (0.100–0.250 g/dL; F(3,16) = 2.765; *p* = 0.0759) and TG (1.078–1.821 g/dL; F(3,16) = 2.505; *p* = 0.0961).

#### 3.3.3. The Protein Expression of LXRs

The comparison of the expression of LXRα and LXRβ did not reveal significant differences between the groups (F_LXRα_ (3,16) = 2.960; *p* = 0.0638 and F_LXRβ_ (3,16) = 0.900; *p* = 0.4628). Regarding to NDV, HFDV presented increases in the expression of LXRα and LXRβ (26.80% and 21.97%; respectively; *p* < 0.05) [5]. Also, in the animals fed with a normal diet, the METa supplementation did not produce any effects on the expression of LXR (F_LXRα_ (3,16) = 0.301; *p* = 0.8244 and F_LXRβ_ (3,16) = 1.669; *p* = 0.2135).

## 4. Discussion

In this report, the UHPLC-ESI-QTOF-MS analysis reveals the presence of thirty identified compounds, including twelve flavonoids, five terpenes, two fatty acids, three phorbolesters and eight phenolic acids. Among them, six di-caffeoylquinic acids (peaks 2 to 7) were identified, including cynarin. These compounds have been previously reported for their roles as free radical scavengers, antioxidants, lipid peroxidation inhibitors, antitumor agents, and hepatoprotectors [6,7]. The detected antioxidant capacity could be associated with the total content of phenolics in METa. Also, they may contribute to the ILP process as previously reported for other Andean medicinal species [23,24]. One potential explanation for this inhibition could be attributed to the presences of phenolic acids donating hydrogen atoms to lipid radicals, resulting in the formation of more stable lipid derivatives and radicals. These new radicals are less prone to promoting auto-oxidation, contributing to the protective effects against lipid peroxidation [25].

In a recent study, decoctions of *T. absinthiodes*, collected in Chile and Argentina, exhibited potent DPPH scavenging activity (EC_50_: 42–43 µg/mL) and significant ILP (86–88% at 250 µg/mL) [4]. Partitioning studies of METa using Sephadex LH-20 are currently underway to unequivocally identify the compounds responsible for ILP. These studies aim to shed light on the potential mechanisms of action of these compounds.

The administration of METa in animals fed with a high-fat diet revealed promising results. The supplementation with METa in any dose does not change the BW gain of the animals or the food intake, in agreement with previous reports using polyphenol compounds or a decoction of *T. absinthioides* in rodents fed with a high-fat diet [5,26].

The dose of METa_500_ reduced the levels of TC. Also, all the doses increased the levels of HDL-c but did not modify the levels of LDL-c and TG. These results are in agreement with our previous report [5], where a decoction of *T. absinthioides* improved the levels of TC and HDL-c after 4 and/or 6 weeks of supplementation. However, it does not appear to significantly affect the levels of TG during the same period [5].

Compounds such as chlorogenic acid, caffeoylquinic acid, and di-caffeoylquinic acids, found in the Asteraceae family and elsewhere, have demonstrated the ability to induce hypocholesterolemic effects in vitro [27]. Also, cynarin has potential effects on choleretic and cholesterol lowering [28]. The flavonoids promote an increase in fecal sterols that in turn leads to a decrease in the absorption of dietary cholesterol [29]. In addition, the consumption of flavonoids increases the levels of HDL-c, which removes the cholesterol from peripheral tissues to the liver for catabolism and excretion [30]. Moreover, flavonoids and polyphenols may contribute to the hypolipidemic activity by increasing the cholesterol metabolism and modulating the enzymes involved in this process [31]. Dietary supplementation with plant sterols may also reduce the levels of TC. Sitosterol, stigmasterol, campesterol, brasicasterol and ergosterol have a similar chemical structure to cholesterol and are poorly absorbed in the intestine. These plant sterols reduce the absorption of cholesterol, displacing it from bile micelles [32].

Several compounds, like diterpenes, phenolic acids and stanols (phytosterols/phytostanols), present in *T. absinthioides* are capable of modulating the activity of LXRs [15,33,34]. Also, caffeoylquinic acid can modulate the hepatic expression of LXRα and improve the lipid metabolism disorders observed in a high-fat diet model [15]. However, in this model, the supplementation with METa did not modify the expression of LXRs, as previously reported after two weeks of a decoction of *T. absinthioides* supplementation [5].

In summary, the exhaustive UHPLCMS/MS study presented in this work updates the chemical profile of the South American medicinal species *T. absinthioides* and provides relevant information on how one or more components present in the plant could improve oxidative and hypercholesterolemic states. However, the precise mechanism underlying these actions requires further molecular and mechanistic studies. Additional research is necessary to determine if *T. absinthioides* can be considered a new source of beneficial phytocompounds and to extrapolate these effects to humans.

## 5. Conclusions

In this report, thirty compounds, including phenolic acids, phorbolesters, flavones, eudesmane sesquiterpenoids, and fatty acids, were identified in METa using MS spectra and MSn experiment data obtained through UHPLC-ESI-QTOF-MS analysis in negative mode. Among the compounds identified, six di-caffeoylquinic acids may contribute, at least partially, to the demonstrated antioxidant and hypocholesterolemic activities of METa. Although molecular and mechanistic studies are required to elucidate the action pathways, the present report adds relevant information about how one or more components present in this plant can help to handle oxidative stress and hypercholesterolemic states. Additionally, this is the first report that involves the study of a methanolic extract of *T. absinthioides* in an animal model of hypercholesterolemia. While the results are promising, further research is needed to establish whether *T. absinthioides* can be recognized as a new source of beneficial phytocompounds and can extend the observed metabolic effects to humans.

## Figures and Tables

**Table 1:** High resolution UHPLC–PDA–Q-TOF identification of metabolites from METa.

Peak	Tentative Identification	[M-H]^−^	Retention Time (min)	Theoretical Mass (*m/z*)	Measured Mass (*m/z*)	Accuracy (ppm)	Metabolite Type	MS Ions (ppm)
1	Naformiate (internal standard)	C_4_H_2_O_4_	0.22	112.98293	112.98562	3.1	Standard acid	588.8964, 656.8829, 724.8745
2	Di-caffeoyl quinic acid (cynarin) 1,5 di C-QA)	C_25_H_23_O_12_	0.72	515.11952	515.12013	1.99	Phenolic acid	353.0883, (CA) 191.0571
3	Di-caffeoyl quinic acid (3,5 di C-QA)	C_25_H_23_O_12_	0.72	515.11952	515.12113	3.26	Phenolic acid	353.0881, (CA) 191.0573
4	Di-caffeoyl quinic acid (4,5 di C-QA)	C_25_H_23_O_12_	3.16	515.11952	515.12212	5.17	Phenolic acid	353.0872, 191.0571 (QA)
5	Di-caffeoyl quinic acid (1,3 di C-QA)	C_25_H_23_O_12_	5.15	515.11952	515.12095	2.83	Phenolic acid	353.0873, 191.0572 (QA)
6	Di-caffeoyl quinic acid (1,4 di C-QA)	C_25_H_23_O_12_	5.17	515.11952	515.12101	2.95	Phenolic acid	353.0874, 191.0574 (QA)
7	Di-caffeoyl quinic acid (1,5 di C-QA)	C_25_H_23_O_12_	5.21	515.11952	515.11957	0.16	Phenolic acid	353.0872, 191.0575 (QA)
8	Tessaric acid derivative	C_15_H_19_O_2_	3.16	231.13981	231.13905	9.86	Phenolic acid	187.14872
9	Tessaric acid	C_15_H_19_O_3_	8.45	247.13397	247.13383	−0.45	Terpene	205.15975
10	5-Acetyl, 3-hydroxy-4 dihydrocostic acid	C_34_H_47_O_7_	8.75	309.17090	309.16962	3.95	Terpene	291.16019
11	Axillarin	C_17_H_13_O_8_	9.32	345.06068	345.0615	−2.6	Flavone	315.0507, 691.13021 (2M-H), 179.9921, 151.00349
12	3-*O*-Caffeoyl-5-*O*-malonylquinic acid	C_19_H_19_O_12_	9.94	439.08233	439.08363	3.0	Phenolic acid	341.12645
13	gamma Costic acid	C_15_H_21_O_2_	11.02	233.15470	233.15516	1.93	Terpene	
14	Rhamnacin	C_17_H_13_O_7_	11.48	329.06574	329.0666	−2.91	Flavone	315.0462, (M-CH3), 277.1075, 300.05554, 151.0020, 256.03405
15	Hesperetin	C_16_H_13_O_6_	11.93	301.07142	301.07162	−0.42	Flavone	263.12825, 201.05187
16	Isorhamnetin	C_16_H_11_O_7_	12.62	315.05113	315.05153	−2.6	Flavone	299.05616 (M-CH3), 287.0555
17	Irigenin	C_18_H_15_O_8_	13.32	359.07724	359.07665	−1.63	Flavone	341.06717, 317.0666, 299.0643, 112.9847, 179.0761,
18	Europetin 7-*O*-methylmyricetin	C_16_H_11_O_8_	13.84	331.04573	331.04574	0.0	Flavone	329.0656, 315.0513, (M-CH3), 301.0351 (M-2CH3)
19	Cirsiliol	C_17_H_13_O_7_	14.39	329.06664	329.06692	0.92	Flavone	315.0465, (M-CH3), 271.0260, 300.05554, 151.0020, 256.03405
20	Arcapillin	C_18_H_15_O_8_	14.95	359.07754	359.07618	−3.19	Flavone	277.1078, 112.9847, 179.0761,
21	Sakuranetin	C_16_H_13_O_5_	15.35	285.07682	285.07593	−2.96	Flavone	247.0972,
22	Genkwanin	C_16_H_12_O_5_	16.11	283.06123	283.06023	−2.92	Flavone	247.1515, 269.0407, 201.1487
23	Eupatorin (3′,5-Dihydroxy-4′,6,7-trimethoxyflavone)	C_18_H_15_O_7_	16.32	343.08258	343.08117	−3.58	Flavone	329.06662, 271.0255, 165.0193
24	2-Hydroxyoctadecanoic acid	C_18_H_35_O_3_	17.21	299.26085	299.25917	−2.71	Fatty acids	643.3434
25	Eupatorin 5-methyl eter	C_19_H_17_O_7_	17.46	357.08234	357.08232	0.03	Flavone	321.1702
26	Eudesma-2,4(15),11(13)-trien-12-oic acid	C_15_H_19_O_2_	17.55	231.13905	231.13974	12.2	Terpene	161.04478
27	Palmitic acid	C_16_H_31_O_2_	17.72	255.23475	255.23295	7.04	Fatty acids	231.13511
28	Cryptocaryol D	C_40_H_63_O_8_	18.41	585.40081	585.39821	−4.43	Terpene	460.35166
29	4 alpha-Phorbol 12,13-didecanoate	C_40_H_63_O_8_	18.53	671.47877	671.47151	−3.67	Phorbolester	586.40293, 584.44304, 491.32338
30	4 alpha-Phorbol 2,13-didecanoate	C_40_H_63_O_8_	18.77	671.47397	671.46911	−7.23	Phorbolester	586.40293, 491.32338
31	4alpha-Phorbol2,12-didecanoate	C_40_H_63_O_8_	18.98	671.47397	671.45284	−5.5	Phorbolester	586.40293, 491.32338

**Table 2:** Content of phenols and flavonoids and antioxidant assays of METa.

Content of Phenols	
Total phenolics (mg GAE/g METa)	251.68 ± 6.80
Flavonoids (mg QE/g METa)	116.34 ± 0.43
**Antioxidant Assays**	
DPPH (IC_50_ in µg METa/mL)	22.89 ± 0.03
FRAP (mg TE/g of METa)	2.30 ± 0.25
TEAC (mg TE/g of METa)	0.85 ± 0.02
Percentage ILP (at 100 µg METa/mL)	61.74 ± 0.41
Percentage ILP catechin (100 µg/mL)	74.01 ± 0.10

## Data Availability

Data are contained within the article.
